# Machine learning-based prediction of COVID-19 diagnosis based on symptoms

**DOI:** 10.1038/s41746-020-00372-6

**Published:** 2021-01-04

**Authors:** Yazeed Zoabi, Shira Deri-Rozov, Noam Shomron

**Affiliations:** Sackler Faculty of Medicine, Tel Aviv University, 6997801 Tel Aviv, Israel

**Keywords:** Respiratory signs and symptoms, Population screening, Epidemiology

## Abstract

Effective screening of SARS-CoV-2 enables quick and efficient diagnosis of COVID-19 and can mitigate the burden on healthcare systems. Prediction models that combine several features to estimate the risk of infection have been developed. These aim to assist medical staff worldwide in triaging patients, especially in the context of limited healthcare resources. We established a machine-learning approach that trained on records from 51,831 tested individuals (of whom 4769 were confirmed to have COVID-19). The test set contained data from the subsequent week (47,401 tested individuals of whom 3624 were confirmed to have COVID-19). Our model predicted COVID-19 test results with high accuracy using only eight binary features: sex, age ≥60 years, known contact with an infected individual, and the appearance of five initial clinical symptoms. Overall, based on the nationwide data publicly reported by the Israeli Ministry of Health, we developed a model that detects COVID-19 cases by simple features accessed by asking basic questions. Our framework can be used, among other considerations, to prioritize testing for COVID-19 when testing resources are limited.

## Introduction

The novel coronavirus disease 2019 (COVID-19) pandemic caused by the SARS-CoV-2 continues to pose a critical and urgent threat to global health. The outbreak in early December 2019 in the Hubei province of the People’s Republic of China has spread worldwide. As of October 2020, the overall number of patients confirmed to have the disease has exceeded 39,500,000, in >180 countries, though the number of people infected is probably much higher. More than 1,110,000 people have died from COVID-19^1^.

This pandemic continues to challenge medical systems worldwide in many aspects, including sharp increases in demands for hospital beds and critical shortages in medical equipment, while many healthcare workers have themselves been infected. Thus, the capacity for immediate clinical decisions and effective usage of healthcare resources is crucial. The most validated diagnosis test for COVID-19, using reverse transcriptase polymerase chain reaction (RT-PCR), has long been in shortage in developing countries. This contributes to increased infection rates and delays critical preventive measures.

Effective screening enables quick and efficient diagnosis of COVID-19 and can mitigate the burden on healthcare systems. Prediction models that combine several features to estimate the risk of infection have been developed, in the hope of assisting medical staff worldwide in triaging patients, especially in the context of limited healthcare resources. These models use features such as computer tomography (CT) scans^2–6^, clinical symptoms^7^, laboratory tests^8,9^, and an integration of these features^10^. However, most previous models were based on data from hospitalized patients, thus are not effective in screening for SARS-CoV-2 in the general population.

The Israeli Ministry of Health publicly released data of all individuals who were tested for SARS-CoV-2 via RT-PCR assay of a nasopharyngeal swab^11^. During the first months of the COVID-19 pandemic in Israel, all diagnostic laboratory tests for COVID-19 were performed according to criteria determined by the Israeli Ministry of Health. While subject to change, the criteria implemented during the study period included the presence and severity of clinical symptoms, possible exposure to individuals confirmed to have COVID-19, certain geographical areas, and the risk of complications if infected^12^. Except for a small minority who were tested under surveys among healthcare workers, all the individuals tested had indications for testing^13^. Thus, there was no apparent referral bias regarding the vast majority of the subjects in the dataset used in this study; this contrasts with previous studies, for which such bias was a drawback^14^. In addition, all negative and positive COVID-19 cases this dataset were confirmed via RT-PCR assay^11^.

In this paper, we propose a machine-learning model that predicts a positive SARS-CoV-2 infection in a RT-PCR test by asking eight basic questions. The model was trained on data of all individuals in Israel tested for SARS-CoV-2 during the first months of the COVID-19 pandemic. Thus, our model can be implemented globally for effective screening and prioritization of testing for the virus in the general population.

## Results

### Baseline model

**Fig. 1:**
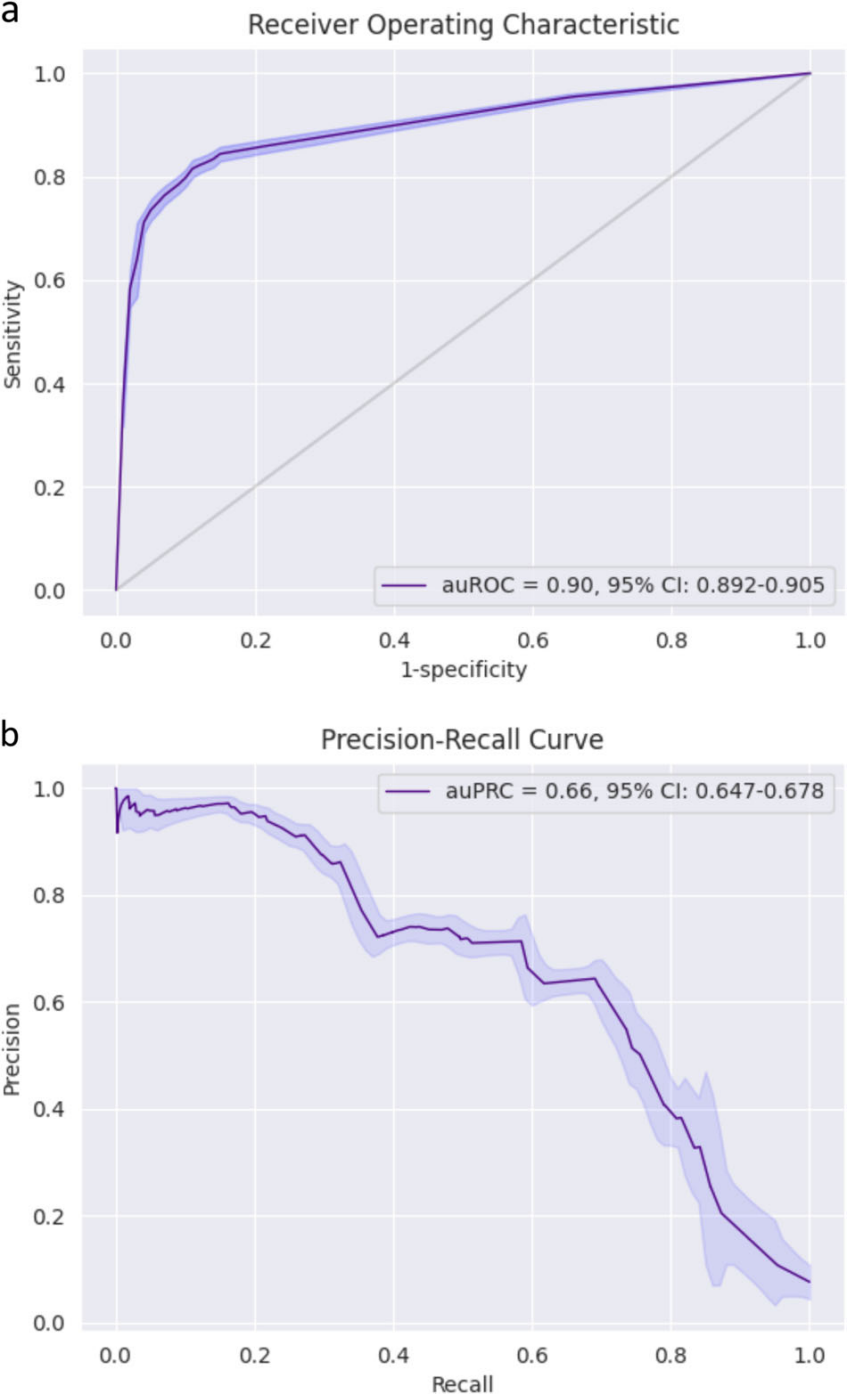
Model performance. **a** ROC curves of the predictive model on the prospective test set. The light band around the curve represents pointwise 95% confidence intervals derived by bootstrapping. **b** A plot of the precision (positive predictive value, PPV) against the recall (sensitivity) of the predictor for different thresholds. The light band around the curve represents pointwise 95% confidence intervals derived by bootstrapping.

For the prospective test set, the model predicted with 0.90 auROC (area under the receiver operating characteristic curve) with 95% CI: 0.892–0.905 (Fig. 1a). Using predictions from the test set, the possible working points are: 87.30% sensitivity and 71.98% specificity, or 85.76% sensitivity and 79.18% specificity. Figure 1b presents the PPV (positive predictive value) of a COVID-19 diagnosis against sensitivity, with auPRC (area under the precision-recall curve) of 0.66 with 95% CI: 0.647–0.678. The metrics from all ROC curves appearing in this study were calculated and are found in a supplementary excel file (Supplementary Data 1).

**Fig. 2:**
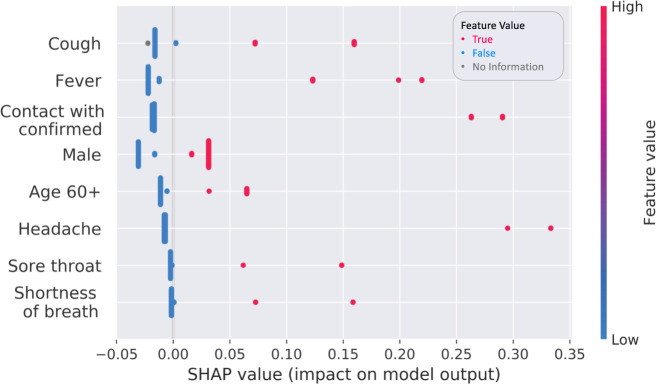
Important features. SHapley Additive exPlanations (SHAP) beeswarm plot for predicting COVID-19 diagnosis, showing SHAP values for the most important features of the model. Features in the summary plots (*y*-axis) are organized by their mean absolute SHAP values. Each point corresponds to an individual person in the study. The position of each point on the x-axis shows the impact that feature has on the classifier’s prediction for a given individual. Values of those features (i.e., fever) are represented by their color.

Ranking of the most important features of the model are summarized in Fig. 2. Presenting with fever and cough were key to predicting contraction of the disease. As expected, close contact with an individual confirmed to have COVID-19 was also an important feature, thus corroborating the disease’s high transmissibility^15^ and highlighting the importance of social distancing. In addition, male sex was revealed as a predictor of a positive result by the model, concurring with the observed sex bias^16,17^.

### Training using unbiased features

The data that were reported by the Israeli Ministry of Health has limitations and biases. For instance, symptom reporting was more comprehensive among those who tested positive for COVID-19, and validated with a directed epidemiological effort^13^. Thus, mislabeling of symptoms among those who tested negative for COVID-19 is expected. This is reflected in the proportion of persons who were COVID-19 positive from the total number of individuals who were positive for each symptom. Accordingly, we identified features with biased reporting (headache 96.2%, sore throat 92.3% and shortness of breath 92.4%) and symptoms with balanced reporting (cough 27.4% and fever 45.9%). Mislabeling of symptoms may also arise from an underestimation and underreporting of symptoms among persons who tested negative.

**Fig. 3:**
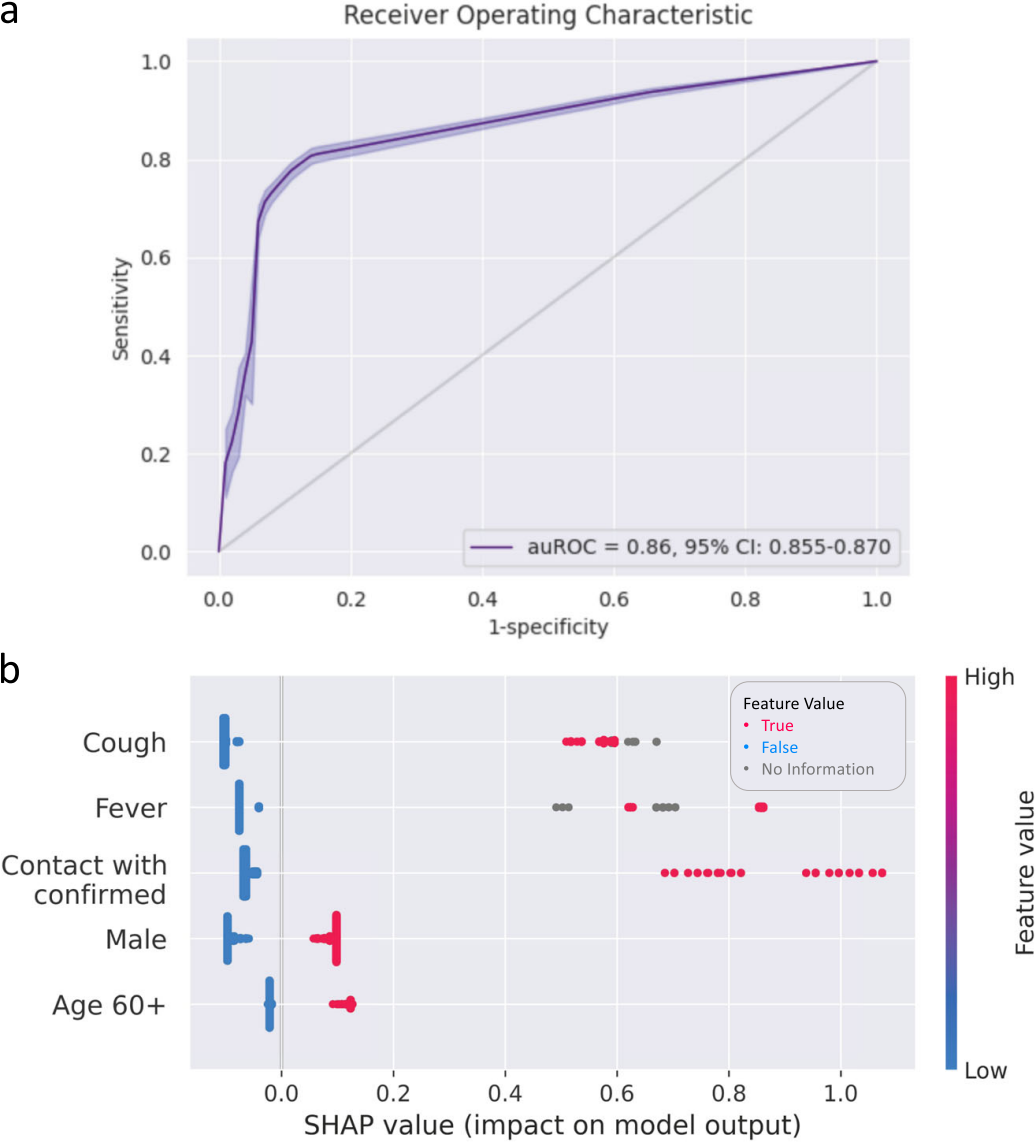
Performance using only balanced features. **a** ROC curve and **b** SHAP beeswarm plot for the prospective test set through training, using only balanced features.

If we train and test our model while filtering out symptoms of high bias in advance, we obtain an auROC of 0.862, with a slight change in the SHAP (SHapley Additive exPlanations) summary plot (Fig. 3).

## Discussion

The unique pathogenesis mechanisms of SARS-Cov-2, and the related spectrum of symptoms are the subject of many ongoing studies. The model we built provides initial COVID-19 test screening based on simple clinical signs and symptoms. Improving clinical priorities may lower the burden currently faced by health systems^18^, by facilitating optimized management of healthcare resources during future waves of the SARS-Cov-2 pandemic. This is especially important in developing countries with limited resources.

**Fig. 4:**
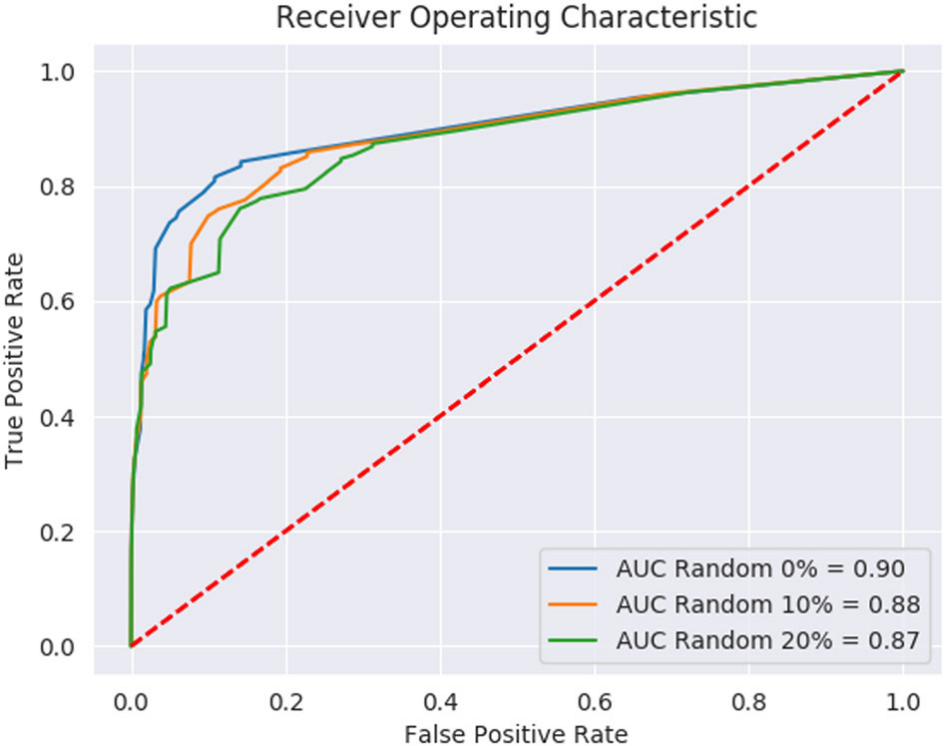
Performance on stimulated test sets. ROC curves showing the performance of the model on stimulated test sets, in which we randomly selected negative reports for all five symptoms at a time and substituted them with blank values. The ROC curve for the original test set is shown in blue. The orange and green curves are ROC curves for randomly substituting 10% and 20%, respectively, of the negative values for all five symptoms.

This research is not without shortcomings. We relied on the data reported by the Israeli Ministry of Health, which has limitations, biases and missing information regarding some of the features. For example, for patients labeled as having had contact with a person confirmed to have COVID-19, additional information such as the duration and location (indoors/outdoors) of the contact was not available. Some symptoms (such as lack of smell and taste) were identified as being very predictive of a COVID-19 infection by previous studies^19^, but were not recorded by the Israeli Ministry of Health. We showed that training and testing a model while filtering out symptoms of high bias in advance still achieved very high accuracy. We also note that all the symptoms were self-reported, and a negative value for a symptom might mean that the symptom was not reported. It is therefore important to assess the model’s performance in the circumstance that more values are unreported or missing rather than with negative values. To simulate a less biased condition, in our prospective test set, we randomly selected negative reports of all five symptoms at a time, and removed the negative values. When applied to these simulated test sets, the model still showed promising results (Fig. 4), thus reinforcing our confidence in the model.

While differences in reporting symptoms is a possible limitation of our model, all the persons tested (except for a small minority who were tested under surveys of healthcare workers) had indications for testing^13^. This implies that there was no referral bias for the vast majority of the subjects in this dataset. The main symptoms in the Israeli Ministry of Health guidelines were cough and fever, and we believe that these symptoms are hard to miss even in those who were negative to SARS-Cov-2. Moreover, we assume that the relatively large sample size helped overcome biases related to the COVID-19-negative group.

We highlight the need for more robust data to complement our framework, while also acknowledging that self-reporting of symptoms is always subject to bias. As the COVID-19 pandemic progresses, ongoing recording and sharing of robust data between public organizations and the scientific community are crucial. In parallel to increasing understanding of the contribution of various symptoms to diagnosing the disease, additional symptoms might be integrated into future models.

In conclusion, based on nationwide data reported by the Israeli Ministry of Health, we developed a model for predicting COVID-19 diagnosis by asking eight basic questions. Our framework can be used, among other considerations, to prioritize testing for COVID-19 when testing resources are limited. In addition, the methodology presented in this study may benefit the health system response to future epidemic waves of this disease and of other respiratory viruses in general.

## Methods

### Setting and study data

The Israeli Ministry of Health publicly released data of individuals who were tested for SARS-CoV-2 via RT-PCR assay of a nasopharyngeal swab^11^. The dataset contains initial records, on a daily basis, of all the residents who were tested for COVID-19 nationwide. In addition to the test date and result, various information is available, including clinical symptoms, sex and a binary indication as to whether the tested individual is aged 60 years or above. Based on these data, we developed a model that predicts COVID-19 test results using eight binary features: sex, age 60 years or above, known contact with an infected individual, and five initial clinical symptoms.

The training-validation set consisted of records from 51,831 tested individuals (of whom 4769 were confirmed to have COVID-19), from the period March 22th, 2020 through March 31st, 2020. The test set contained data from the subsequent week, April 1st through April 7th (47,401 tested individuals, of whom 3624 were confirmed to have COVID-19). The training-validation set was further divided to training and validation sets at a ratio of 4:1 (Table 1).

**Table 1:** Characteristics of the dataset and the features used by the model in this study.

(#) Feature	Total *n* = 99,232		COVID-19 negative *n* = 90,839		COVID-19 positive *n* = 8393	
	*n*	%	*n*	%	*n*	%
(1) Sex						
Male	50,350	50.74	45,545	50.1	4805	57.2
Female	48,882	49.26	45,294	49.8	3588	42.7
(2) Age 60+						
True	15,279	15.4	13,619	14.9	1660	19.7
False	83,953	84.6	77,220	85	6733	80.2
(3) Cough						
True	14,768	14.88	10,715	11.8	4053	48.2
False	84,223	84.87	79,909	87.9	4314	51.4
(4) Fever						
True	8122	8.18	4387	4.83	3735	44.5
False	90,868	91.5	86,237	94.9	4631	55.1
(5) Sore throat						
True	1273	1.28	96	0.11	1177	14
False	95,062	95.8	88,059	96.9	7003	83.4
(6) Shortness of breath						
True	930	0.94	71	0.08	859	10.2
False	95,405	96.14	88,084	96.9	7321	87.2
(7) Headache						
True	1799	1.81	68	0.07	1731	20.6
False	94,536	95.27	88,087	96.9	6449	76.8
(8) Known contact with an individual confirmed to have COVID-19						
True	5507	5.55	1455	1.6	4052	48.2
False	93,725	94.45	89,384	98.4	4341	51.8

The following list describes each of the dataset’s features used by the model:

A. Basic information:
  1. Sex (male/female).
  2. Age ≥60 years (true/false)
B. Symptoms:
  3. Cough (true/false).
  4. Fever (true/false).
  5. Sore throat (true/false).
  6. Shortness of breath (true/false).
  7. Headache (true/false).
C. Other information:
  8. Known contact with an individual confirmed to have COVID-19 (true/false).

### Development of the model

Predictions were generated using a gradient-boosting machine model built with decision-tree base-learners^20^. Gradient boosting is widely considered state of the art in predicting tabular data^21^ and is used by many successful algorithms in the field of machine learning^22^. As suggested by previous studies^23^, missing values were inherently handled by the gradient-boosting predictor^24^. We used the gradient-boosting predictor trained with the LightGBM^25^ Python package. The validation set was used for early stopping^26^, with auROC as the performance measure.

To identify the principal features driving model prediction, SHAP values^27^ were calculated. These values are suited for complex models such as artificial neural networks and gradient-boosting machines^28^. Originating in game theory, SHAP values partition the prediction result of every sample into the contribution of each constituent feature value. This is done by estimating differences between models with subsets of the feature space. By averaging across samples, SHAP values estimate the contribution of each feature to overall model predictions.

### Evaluation of the model

The model was scored on the test set using the auROC. In addition, plots of the PPV against the sensitivity (precision–recall curve) were drawn across different thresholds. Metrics were calculated for all the thresholds from all the ROC curves, including sensitivity, specificity, PPV and negative predictive value, false-positive rate, false-negative rate, false discovery rate and overall accuracy. Confidence intervals (CI) for the various performance measures were derived through resampling, using the bootstrap percentile method^29^ with 1000 repetitions.

### Ethics declarations

The Tel-Aviv University review board (IRB) determined that the Israeli Ministry of Health public dataset used in this study does not require IRB approval for analysis. Therefore, the IRB determined that this study is exempted from an approval.

### Reporting summary

Further information on research design is available in the Nature Research Reporting Summary linked to this article.

## Supplementary information

Descriptin of Additional Supplementary Files

Supplementary Data 1

Reporting Summary

## Data Availability

All the data used in this study were retrieved from the Israeli Ministry of Health website^11^. The dataset was downloaded, translated into English, and can be accessed at: https://github.com/nshomron/covidpred.

## Code availability

The model hyperparameters and the analytic code of the model required to reproduce the predictions and the results are available at: https://github.com/nshomron/covidpred.
