# Supplementary material for: Machine learning-based prediction of COVID-19 diagnosis based on symptoms
Source: NPJ Digit Med. 2021 Jan 4;4:3. doi: 10.1038/s41746-020-00372-6 (PMC7782717; doi:10.1038/s41746-020-00372-6)
Supplement: Supplementary file 1 — Descriptin of Additional Supplementary Files [file 41746_2020_372_MOESM1_ESM.pdf]

Supplementary Data 1 An Excel file containing metrics from all ROC curves appearing in this study shown in their respective sheet.
